# Event-Related Potential Effects of Object Repetition Depend on Attention and Part-Whole Configuration

**DOI:** 10.3389/fnhum.2016.00478

**Published:** 2016-09-23

**Authors:** Angela Gosling, Volker Thoma, Jan W. de Fockert, Alan Richardson-Klavehn

**Affiliations:** ^1^Centre for Cognition and Cognitive Neuroscience, Department of Psychology, Bournemouth UniversityPoole, UK; ^2^School of Psychology, University of East LondonLondon, UK; ^3^Department of Psychology, Goldsmiths, University of LondonLondon, UK; ^4^Memory and Consciousness Research Group, Department of Neurology, Otto-von-Guericke UniversityMagdeburg, Germany

**Keywords:** attention, EEG, ERPs, event-related potentials, object recognition, repetition, view-dependence

## Abstract

The effects of spatial attention and part-whole configuration on recognition of repeated objects were investigated with behavioral and event-related potential (ERP) measures. Short-term repetition effects were measured for probe objects as a function of whether a preceding prime object was shown as an intact image or coarsely scrambled (split into two halves) and whether or not it had been attended during the prime display. In line with previous behavioral experiments, priming effects were observed from both intact and split primes for attended objects, but only from intact (repeated same-view) objects when they were unattended. These behavioral results were reflected in ERP waveforms at occipital–temporal locations as more negative-going deflections for repeated items in the time window between 220 and 300 ms after probe onset (N250r). Attended intact images showed generally more enhanced repetition effects than split ones. Unattended images showed repetition effects only when presented in an intact configuration, and this finding was limited to the right-hemisphere electrodes. Repetition effects in earlier (before 200 ms) time windows were limited to attended conditions at occipito-temporal sites during the N1, a component linked to the encoding of object structure, while repetition effects at central locations during the same time window (P150) were found for attended and unattended probes but only when repeated in the same intact configuration. The data indicate that view-generalization is mediated by a combination of analytic (part-based) representations and automatic view-dependent representations.

## Introduction

A central question in the study of visual cognition concerns the nature of the mental representations mediating the recognition of familiar objects. To investigate this issue, many experiments measure priming: Objects are typically recognized faster and more accurately when they are seen a second time (Bartram, 1976). Priming is usually greatest when a repeated object is shown in the same view and decreases when the repeated object is shown in a different view, for example, when it is rotated in the picture plane (see Thoma et al., 2004; Peissig and Tarr, 2007, for brief reviews). Recently, however, priming studies have shown that view-dependent priming effects are moderated by visual attention: Stankiewicz et al. (1998) and Thoma et al. (2004) have shown that whereas attended objects prime themselves in identical and changed views, unattended objects prime themselves in identical views only. The current paper examines the potential neural correlates of these results by employing electroencephalogram (EEG) measures of object repetition.

Studies using scalp recorded event-related potentials (ERPs, see Grill-Spector et al., 2006) have been increasingly used to investigate object priming. The excellent temporal resolution of ERPs can track the time course of neural representations associated with the repeated presentation of an object. Importantly, ERP repetition effects can provide a means to distinguish early visuo-perceptual and later cognitive processes that support repetition in the brain and index the properties of neural representations following changes in image parameters between initial and subsequent presentations of an object (Schendan and Kutas, 2003; Grill-Spector et al., 2006)

The earliest repetition-sensitive ERP components (<200 ms post stimulus onset) have been observed following immediate or short lag repetition as sensitive to variation in shape or view between an initial and repeated exposure with an object (Zhang et al., 1997; Doniger et al., 2001; Itier and Taylor, 2002, 2004; Schendan and Kutas, 2003; Henson et al., 2004; Trenner et al., 2004; Martin-Loeches et al., 2005; Soldan et al., 2006)^1^.

Schendan and Kutas (2003) found form- or image-specific ERP repetition effects, sensitive to changes in object view, as an index of early visuoperceptual categorization. The effect was reported as enhanced positive mean amplitude at vertex and fronto-central scalp sites (between 140 and 250 ms post stimulus) as a P150 ERP component. A temporally parallel ERP repetition priming effect has also been observed as an attenuation of an occipital–temporal N1 component (160–190 ms) triggered by immediate and short-lag repeated objects (Henson et al., 2004). More recently Harris et al. (2009) have reported a P150 ERP component sensitive to object-repetition priming that did depend on participants’ explicit memory and was found regardless of whether primes were encoded during deep or shallow processing during initial presentation. The temporal and spatial distribution of the P150 reported by Harris et al. (2009), was similar to that found by Schendan and Kutas (2003), and the authors suggested the P150 is an index of perceptual object priming mechanisms. ERP modulations during the N1/P150 are thought to reflect neural representations that engage perceptual operations such as low-level visual categorization (Schendan and Kutas, 2003), the encoding of an object’s global structure (Eimer, 2000; Doniger et al., 2001; Itier and Taylor, 2002, 2004), and index low-level visual discrimination processes (Vogel and Luck, 2000).

More consistent and long-lasting ERP repetition effects for visually presented objects are reported for time windows after 200 ms post stimulus onset (Grill-Spector et al., 2006). Henson (2003) identified object repetition effects sensitive to the ‘lag’ between the initial and repeated exposure of an object as enhanced negative amplitudes at occipital–temporal sites during latencies of 200–300 post stimulus onset. Repetition-sensitive ERPs observed during the time window 200–300 ms have also been reported as an N250r component or early repetition effect (Martin-Loeches et al., 2005) evident as an enhanced negative-going mean amplitude over right inferior occipito-temporal regions following the immediate repetition of faces (Schweinberger et al., 2004; Zimmermann and Eimer, 2013), and at left posterior scalp sites to repeated words (Pickering and Schweinberger, 2003), and with a bilateral posterior scalp distribution to immediately repeated objects (Martin-Loeches et al., 2005; Gruber and Muller, 2006; Scott et al., 2006).

The domain-sensitivity reflected in the functional characteristics and neural distribution of the N250r component is consistent with the activation of cortical generators that operate as perceptual representation sub-systems, for example, a structural description system (SDS), word form descriptions, and face recognition units (Zhang et al., 1997; Martin-Loeches et al., 2005). Martin-Loeches et al. (2005) investigated ERP priming effects using either pictures of objects and faces or their names. The authors observed an enhanced occipital–temporal N250r (200–300 ms) for both face and object images, thought to reflect the comparison between structural representations with modality specific (pre-semantic) stored representations. Zhang et al. (1997) reported ERP repetition effects with a similar spatial and temporal distribution to the N250r as a visual memory potential (VMP; 220–260 ms post stimulus onset) with a right lateral posterior maximum enhanced for same-view repeated objects as compared with novel ones (Zhang et al., 1997). Zhang et al. (1997) propose that the VMP reflects the output of neural generators involved in a SDS and that these underlie the constancy of vision despite the infinite views that an object can input to the retinae (Zhang et al., 1997). Later ERP repetition effects reported as an N400 component are considered to reflect access to semantic and conceptual levels of object knowledge (Eddy et al., 2006).

Taken together, the time course of reported ERP repetition effects provide compelling evidence for both early visuo-perceptual neural operations that underlie the rapid categorization of objects in image sensitive views (P150) and later neural representations that code abstract structural descriptions that can account for the constancy of object recognition across numerous views and viewing conditions (N250r). In general, observations indicate that the magnitude of ERP repetition effects depends on the amount of view similarity between initial and subsequent presentation of an object (Zhang et al., 1997; Schendan and Kutas, 2003) and add support to theories proposing that object recognition is mediated by view-specific representations (Schendan and Kutas, 2003), possibly by some type of interpolation across several 2D views of an object (Ullman, 1989, 1998; Poggio and Edelman, 1990; Bülthoff and Edelman, 1992; Logothetis et al., 1994; Tarr and Gauthier, 1998), or via a distributed neural representation across view-tuned neurons (Perrett et al., 1998). However, other studies suggest that object constancy is not achieved via stored object views but by extracting object parts and their spatial relations (such as when the handle part of a cup is coded as ‘side-attached’ to the main cylinder, Biederman, 1987). Although structural description models have some robust support (Biederman and Gerhardstein, 1993; Biederman and Bar, 1999) they cannot account for the apparent speed and automaticity of object processing (Oram and Perrett, 1992; Intraub, 1999; Fabre-Thorpe et al., 2001) because binding objects’ parts and spatial relations would require time (Hummel, 2001) and attention (Hummel and Biederman, 1992).

**FIGURE 1:**
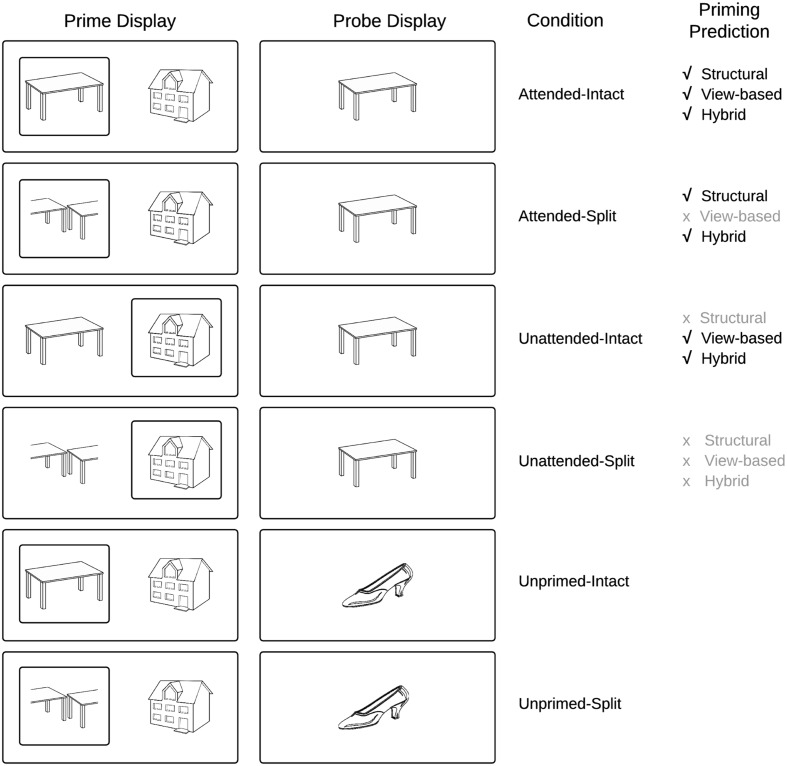
**Schematic overview of experimental conditions and predictions derived from current models of object recognition**.

To jointly address these properties of object recognition, a hybrid model has been proposed that accounts for both the rapid image dependent representation of visual objects but also neural mechanisms that can account for the constancy of visual recognition across changes in input image parameters across initial and repeated exposure of an item (Hummel and Stankiewicz, 1996; Hummel, 2001). In this model, visual attention is necessary in an ‘analytic’ route to bind parts and spatial relations to form a relatively view-independent representation of an object’s shape (Hummel and Biederman, 1992), while a fast process establishes a ‘holistic’ representation which is independent of visual attention (see Hummel, 2001, for details). Unlike in view-based theories Hummel’s hybrid model predicts that attended objects prime themselves regardless of the viewpoint they are shown in (**Figure 1**). In addition, unlike structural description theories the hybrid model predicts priming for unattended objects if they are shown in the same view across repetitions (Stankiewicz and Hummel, 2002). These predictions have been tested and largely confirmed behaviorally (Stankiewicz et al., 1998; Thoma et al., 2004; Thoma and Davidoff, 2007), in neuropsychological studies (Davidoff and Warrington, 1999; Forti and Humphreys, 2005), and more recently, in neuro-imaging studies (Thoma and Henson, 2011). However, so far no EEG study has tested the model’s predictions.

We investigate in this study, ERP components that underlie visual object constancy by examining neural repetition effects while manipulating view-changes between prime and probe trials. The current study followed the approach of Thoma and Henson (2011) and presented objects either as an intact (whole) line-drawing, or as a vertically or horizontally split version of itself (**Figure 1**) in which the halves swap locations. This manipulation, which affects the holistic configuration while leaving the part-based description largely intact, has been shown to distinguish between part-based and view-based representations (Thoma et al., 2004; Hayward et al., 2010)^2^.

**FIGURE 2:**
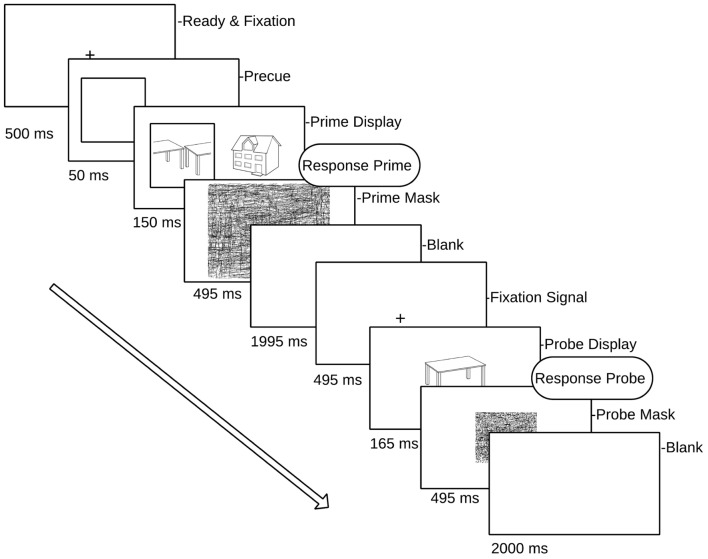
**Schematic overview of a single prime–probe trial pair**. Prime (attended) targets are displayed in a square (pre-cued) randomly to left or right of fixation cross. Participants responded to each (attended) prime and the subsequent probe.

The second important factor in testing the hybrid model concerns visual attention. The hybrid model predicts that repetition effects should occur for probes following attended intact and split objects, whereas probe objects following unattended prime images should show repetition effects only in intact views. This prediction has not yet been tested directly using ERP repetition effects. Neumann et al. (2011) found repetition effects (in the 200–300 ms time window) for faces but not houses or hands, independent of an attentional (perceptual load) manipulation. This and other studies on object repetition effects often employ paradigms in which attended and unattended objects are not spatially separated, a manipulation of attention that may not be as effective as spatial cueing in minimizing attentional slippage to an unattended object (Lachter et al., 2004; Henson and Mouchlianitis, 2007). Therefore, similar to the behavioral studies of Thoma et al. (2004), selective visual attention in the current study was manipulated by spatially cueing attention to one of two briefly displayed, spatially separated objects (**Figure 2**).

The present study examined ERP repetition effects for an intact object image (the probe object) following immediately preceding images of either the same or a different object (the prime object). The variable of prime view (intact vs. split) was factorially crossed with its cued location (cued vs. uncued), a manipulation that has been shown to successfully direct spatial attention to the cued object (Stankiewicz et al., 1998) the terms ‘attended’ and ‘unattended’ refer to primes that were either ‘cued’ or ‘uncued.’ Each object appeared only in one prime–probe trial pair across the experiment, thereby guarding against contamination of repetition effects due to long-term priming (Thoma and Henson, 2011). In order to minimize EEG artifacts induced by speech-related movement, we employed a covert naming task (subvocal naming accompanied by a simultaneous button press, see Martinovic et al., 2008). The key press associated with the subvocal naming of the prime and probe image provided a behavioral response time measure of priming. The sensitivity of this task to neural object repetition effects and behavioral priming has been shown previously (Thoma and Henson, 2011).

To our knowledge this study is the first to investigate the predictions of the hybrid model (Hummel, 2001) using ERP repetition effects. Our predictions for repetition effects focus on two time windows: before 200 ms (N1/P150) and between 220 and 300 ms (N250r/VMP) after probe-object onset. We also limit our analysis to occipital–temporal posterior electrodes and fronto-central and central sites, as employed in previous work (Zhang et al., 1997; Schendan and Kutas, 2003; Henson et al., 2004; Martin-Loeches et al., 2005). According to the hybrid model (Hummel, 2001), ERP repetition effects will be observed for repeated objects, but in the case of split prime objects only when they were attended, as shown in previous behavioral work (Thoma et al., 2004), and also in a more recent fMRI study (Thoma and Henson, 2011) that reports priming for unattended objects only when seen in a repeated view. On this basis, the hybrid model makes the novel prediction that unattended prime objects will elicit ERP repetition effects only when the prime object is intact (same repeated view), whereas attended prime objects will elicit ERP repetition effects both when the prime object is intact and split (Hummel, 2001).

The literature reviewed previously broadly suggests that ERP repetition effects elicited prior to 200 ms post stimulus onset (P150) are sensitive to changes in image input parameters and present only when objects are repeated in the same view, whilst later ERPs following 200 ms (N250r) reflect neural representations involved in object constancy and the representation of invariant structural descriptions. An implicit property of the hybrid model is that holistic representations are generated fast, whereas an analytic representation takes more time to be established, because it relies on synchronized firing of separate units coding parts belonging to the same object. Therefore, following the hybrid model, we predicted that if the current paradigm detects very early repetition effects for objects (within 200 ms of probe onset, i.e., N1/P150), these would be view-specific and independent of attention (as found in the fMRI study of Thoma and Henson, 2011, for dorsal regions of interest) **Figure 1** depicts the priming predictions derived from common object recognition models. **Figure 2** presents a schematic overview of the prime–probe trial procedure.

## Materials and Methods

### Participants

Twenty-nine paid volunteers at Goldsmiths University of London gave written informed consent prior to taking part in the study. Four participants were excluded from analyses due to an insufficient number of trials that remained for averaging after EEG artifact rejection. The remaining 25 participants (16 female) were 20–39 years old (mean age 25.7 years), were all right-handed, had either normal or corrected to normal vision and were native English speakers. The Ethics Committee of the Department of Psychology at Goldsmiths approved the experimental procedures.

### Stimuli and Procedure

The stimulus set consisted of 616 asymmetrical black and white line drawings of familiar objects derived from different sources (Snodgrass and Vanderwart, 1980; Cycowicz et al., 1997). For each image a split counterpart was constructed by dissecting along the main axis of elongation either vertically or horizontally and transposing each half to the opposite side of the object space. Image transformations were performed in Adobe Photoshop CS3 (**Figure 2**). Stimuli were presented on a CRT monitor with the timing set to synchronize with screen refreshes. E-Prime 1.1 (Psychology Software Tools, Pittsburgh, PA, USA) was used for stimulus presentation and behavioral response collection. Stimuli were presented at a viewing distance of 100cm against a light gray background and were standardized in size such that they subtended 4° of visual angle (which meant that centrally presented probe objects fell in the foveal area).

Probe objects were always shown in an intact configuration. The relationship between the prime and the probe display was manipulated to produce six conditions: probe objects could be preceded either by a prime display containing the probe object (attended and intact, attended and split, unattended and intact, or unattended and split), or by a prime display containing only different objects to the probe object (again, the attended prime object could be either intact or split). The resulting six experimental conditions were therefore: Attended-Intact, Unattended-Intact, Unprimed-Intact, and Attended-Split, Unattended-Split, Unprimed-Split.

The experiment was divided into 11 blocks, comprising one practice block and 10 experimental blocks. Each block consisted of 24 prime/probe trial presentations (a total of 56 objects in each block, each block comprising four random of each condition). The allocation of stimuli to conditions was fully counterbalanced across participants, and the order of priming conditions was randomized within blocks, as was the presentation of attended (cued) objects to the left or right of fixation in each prime display. Each trial consisted of a prime and a probe display. The prime display began with a fixation cross (500 ms) followed by a cueing square which subtended 4.45° × 4.45° visual angle. The cue was presented to the left or the right of fixation for 50 ms. The center of the cue was 4° from the mid-point of the screen. Next, two objects were presented either side of fixation, and one of these appeared inside the cue square (attended object) while the other appeared on the uncued side (unattended object). Neither of the object images exceeded 4° degrees of visual angle. Both the attended and unattended prime stimuli remained on the screen for 150 ms followed by a random line mask subtending the whole screen (495 ms). Following an inter-stimulus interval (1995 ms blank screen) and fixation cross (495 ms), a single probe object was presented for 165 ms (see **Figure 2** for a schematic of the prime/probe sequence), followed by a mask (495 ms). The participant’s task was to covertly name the cued prime object and then the probe object, by making a button press to coincide with their silent naming. Trials in which participants did not recognize either the prime or the probe object (and no button press was recorded) were excluded from the analyses.

### EEG Recording

Electroencephalogram was recorded from 64 (Ag/AgCl) electrodes mounted in an elastic cap distributed over the head surface according to the extended 10-20 EEG system (Oostenveld and Praamstra, 2001) with a BioSemi Active-Two amplifier system (BioSemi, Amsterdam, The Netherlands). The placement of electrodes included midline sites with FPz, AFz, Fz, FCz, Cz, CPz, Pz, POz, Oz, and Iz electrodes; Fp1, AF3, AF7, F1, F3, F5, F7, FC1, FC3, FC5, FT7, C1, C3, C5, T7, CP1, CP3, CP5, TP7, P1, P3, P5, P7, P9, PO3, PO7, and O1 electrodes in the left hemisphere; and the corresponding even-numbered recording sites in the right hemisphere. To monitor eye movements and blinks the horizontal and vertical electro-oculogram (EOG) were recorded. EEG and EOG recordings were sampled at 512 Hz with a bandpass of DC-67Hz (bandwidth 3Db). Two additional electrodes (CMS-Common Mode and DRL-Driven Right Leg) were used as reference and ground^3^, signals were re-referenced oﬄine using an average reference. Using Brain Vision Analyzer the EEG was filtered oﬄine using a high pass filter of 0.1 Hz and low pass filter of 40 Hz. EEG was epoched from 100 ms before to 550 ms after the onset of probe objects (S2), relative to a 100 ms prestimulus baseline. Epochs with activity exceeding ±30 μv in the HEOG channel reflecting horizontal eye-movements or ±60 μv at FPz (indicating eye blinks or vertical eye-movements) were excluded from analysis, as were epochs with voltages exceeding ±80 μv at any other electrode. Following artifact rejection, participants’ average ERPs were computed for intact probe objects quantified separately on the basis of the preceding prime condition (**Figure 2**). Resulting grand average ERPs were derived for each of the six experimental conditions. Importantly, to reiterate, all probe objects were presented in an intact configuration and only trials on which a response was collected were retained for analyses.

### Data Analyses

Event-related potential analyses were carried out on the probe trials only. We first assessed the impact of object repetition, followed by an analysis investigating effects of attention and configuration. ERP analyses were focused on those electrodes and time intervals that have been associated with ERP repetition priming in previous studies at occipital–temporal posterior electrodes and at fronto-central sites (Schweinberger et al., 1995; Schendan and Kutas, 2003; Henson et al., 2004; Martin-Loeches et al., 2005). These encompassed the time windows associated with the N1 (140–180 ms) and N250r (220–300 ms) components, which were quantified as ERP mean amplitude values computed from posterior electrode pairs P7/P8 and PO7/PO8. Accompanying mean amplitudes at fronto-central sites (FCz and Cz) were also quantified during the P150 (140–180 ms Schendan and Kutas, 2003).

Initial repeated-measures analyses of variance (ANOVAs) included the factors repetition (attended, unattended, not repeated), hemisphere (left, right), and electrode site (P7/P8, PO7/PO8). There were two separate analyses to establish the presence of reliable ERP repetition priming effects for probes primed either by their intact (same) or split (different) counterparts in the prime display. A second set of analyses focused on attention and configuration effects using difference ERPs computed by subtracting mean amplitudes to unprimed trials from those to the respective (intact and split) primed trials. For analyses at posterior electrodes, resulting difference ERPs were subjected to 2 × 2 × 2 × 2 ANOVAs with factors attention (attended, unattended), configuration (intact, split), hemisphere (left, right) and electrode site (P7/P8, PO7/PO8). For fronto-central analyses, the factor of hemisphere was not included and the site factor was replaced with electrodes FCz/Cz. Greenhouse–Geisser adjustments to the degrees of freedom were applied to analyses of ERPs where appropriate to correct for violations of sphericity.

## Results

### Behavioral Results

Priming was operationalized as the difference in naming latencies for repeated compared with unrepeated probe objects (as measured by the button press, in ms). Trials in which the behavioral response was greater than 2000 ms or shorter than 250 ms were removed from both behavioral and ERP analyses, along with any trials on which a response was not recorded (**Table 1**). There was no significant difference in the number of trials that involved a valid response between the six experimental conditions (all *t*-values < 1.5, all *p*-values > 0.1); see **Table 1** for the percentage of correct responses. An analysis of the behavioral reaction time (RT) data was carried out to assess priming across conditions. RTs to each of the attended primed conditions for intact and split, as well as the unattended intact and split were compared with RTs to unprimed-intact probes and unprimed-split conditions, respectively. Significant priming effects were found for attended intact [*t*(24) = 4.42; *p* < 0.001], attended split [*t*(24) = 4.25; *p* < 0.001], and unattended-intact [*t*(24) = 2.18; *p* = 0.039] conditions. RTs to unattended split objects were not significantly different to those for unprimed objects (*p* > 0.33).

**Table 1:** Mean latencies in milliseconds [standard deviation (SD) in parentheses] and accuracy rates (%) to intact probe stimuli preceded by attended, unattended, or unprimed prime objects in an intact or split configural format.

	Attended		Unattended		Unprimed	
	Intact	Split	Intact	Split	Intact	Split
Latency Mean (*SD*)	502 ms (128)	536 ms (164)	558 ms (169)	566 ms (185)	580 ms (187)	576 ms (188)
Accuracy % (*SD*)	96.20% (4.4)	96.70% (3.6)	95.60% (3.4)	96.50% (3.6)	96.70% (3.2)	96.20% (4.0)

*The percentage of accurate trials was computed in which objects where named and trials involved a valid behavioral response*.

To examine differences in the magnitude of priming effects, further analyses were conducted on the savings in probe response times (for example, the difference between primed and unprimed conditions) using a within-subjects ANOVA with the factors attention (attended/unattended) and configuration (intact/split), analog to previous behavioral work (Thoma et al., 2004). Results revealed main effects of attention [*F*(1,24) = 20.53; *p* < 0.001] and configuration [*F*(1,24) = 5.07; *p* = 0.034] showing that probe RTs to previously attended objects were faster than to previously unattended objects, and probe responses were faster following intact primes than following split prime objects. The interaction between attention and configuration was also significant [*F*(1,24) = 4.53; *p* < 0.05] which reflected the reliable RT difference in priming between intact and split objects for attended conditions [*t*(24) = 3.04; *p* = 0.006] compared with a non-significant difference for unattended conditions (*p* > 0.32). The behavioral results confirm previous findings (Thoma et al., 2004) that the recognition of a probe object is primed by both intact and split versions of the same object that was previously attended, whereas a previously unattended object only primes an intact version of itself.

### Event Related Potential Analyses

**FIGURE 3:**
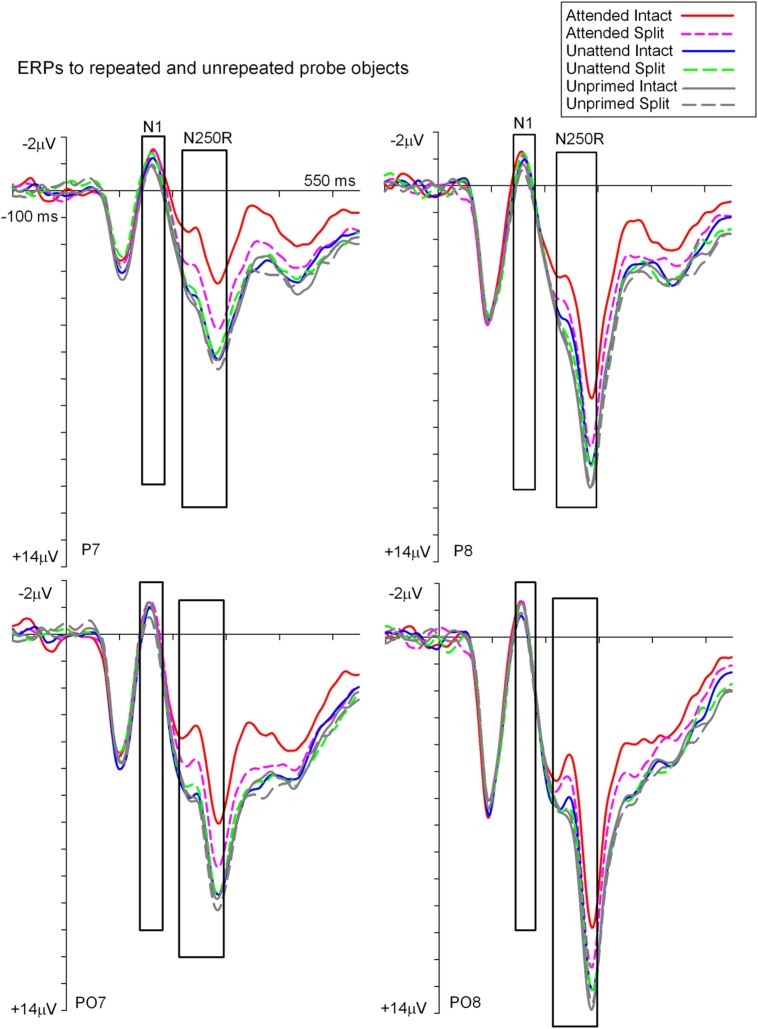
**Grand-average event related potentials to S2 probes measured at lateral posterior electrodes P7/P8 and PO7/PO8 in the time window -100 to 550 ms following stimulus onset**. ERPs are shown separately for each (S2) probe condition on the basis of the preceding (S1) prime trial type; attended, unattended or unprimed in either an intact or split configuration. ERPs during time points of the posterior N1 and N250r components are highlighted in the figure. These time points and electrodes denote ERPs that were the focus of statistical analyses.

**FIGURE 4:**
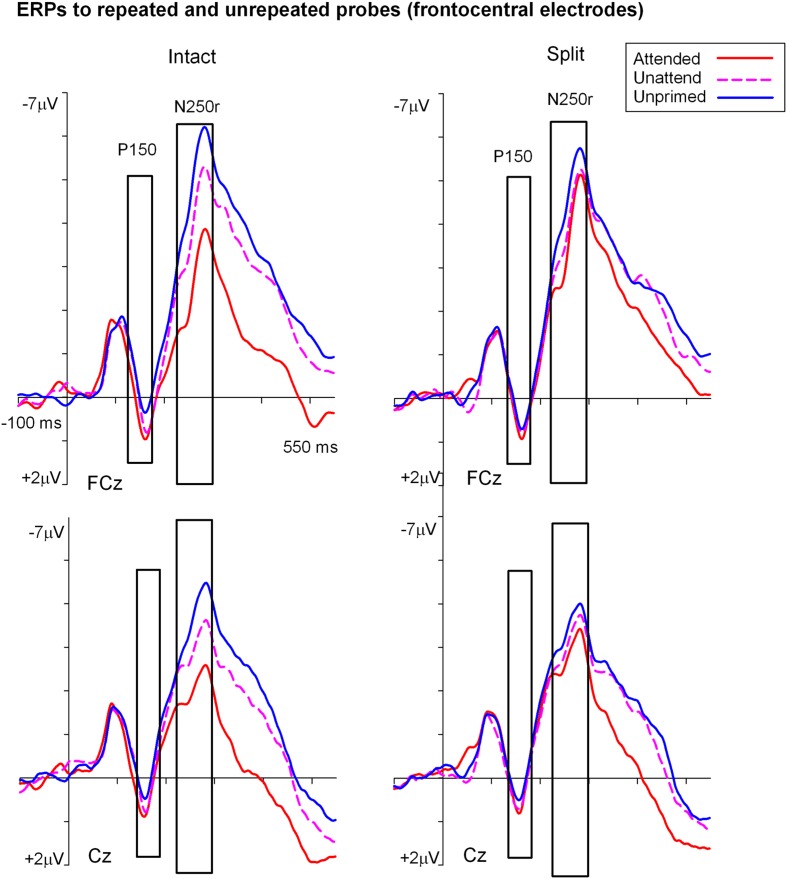
**Grand-average event related potentials elicited at FCz and Cz in the time interval beginning -100 to 550 ms following the onset of (S2) probe objects as a function of the preceding split or intact (S1) prime trial type**. ERPs during the P150 (140–180 ms) were the focus of statistical analyses and are shown highlighted.

**Figures 3** and **4** show grand average ERPs derived from voltages recorded at posterior electrodes P7/P8 and PO7/PO8, and from fronto-central electrodes FCz/Cz, respectively. ERPs were triggered to intact probe objects as a function of the preceding prime trial type: attended, unattended or unprimed in either a split or intact configuration. Statistical evaluation of early ERP repetition effects (<200 ms post stimulus onset) was focused on mean amplitudes recorded at posterior and fronto-central electrodes during the time window 140–180 ms post stimulus onset and corresponding to the N1 and P150 components, respectively.

#### ERP Repetition Effects during N1 (140–180 ms)

**FIGURE 5:**
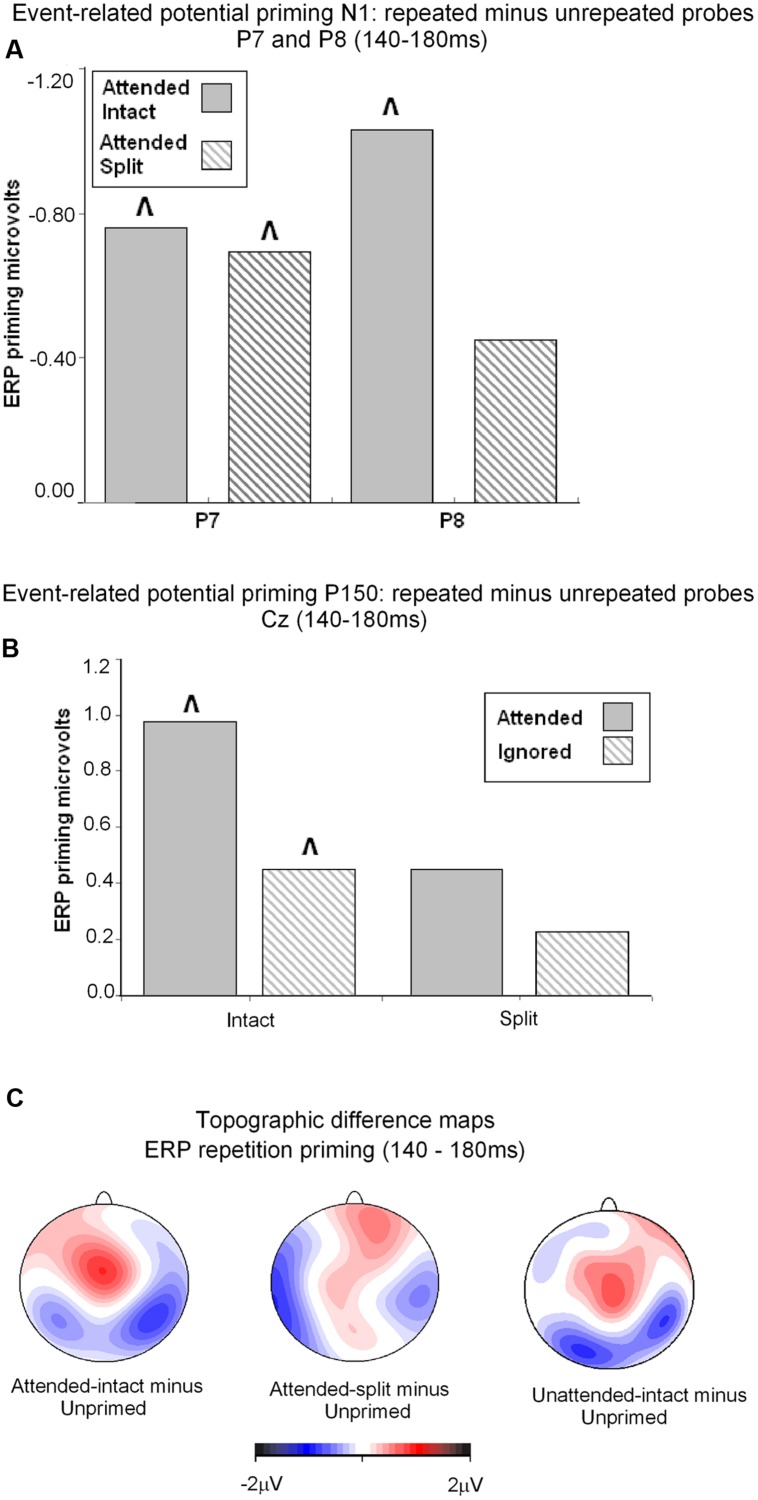
**(A)** Event related potential priming effects during the N1 component (140–180 ms) recorded at posterior electrodes P7 and P8. ERP priming derived by subtracting mean amplitude to attended intact and attended split trials from corresponding intact and split unprimed primes. The symbol ˆ denotes significant ERP priming effects. N1 component ERP priming effects derived to unattended intact and split trials were not reliably different to unprimed new objects. **(B)** ERP mean amplitudes recorded during the latency 140–180 ms (P160) at the vertex (Cz) for (S2) repeated probes as a function of their attended or unattended format on (S1) prime phase in either intact or split format minus mean amplitude to unprimed probes. ˆ denotes significant ERP repetition effects. **(C)** Topographic maps showing significant ERP priming effects obtained during the latency 140–180 ms after S2 (probe) onset by subtracting ERPs to unprimed-intact probes from ERPs to attended-intact, and unprimed-split from attended-split probes and unprimed-intact from unattended-intact. ERPs to unattended split were not significantly different to unprimed-split.

To establish the magnitude and onset of ERP repetition effects at posterior electrode sites during the N1 component (140–180 ms) two repeated measures 3 × 2 × 2 ANOVAs were each focused on N1 mean amplitudes derived from intact and split trials separately. The first of these was a 3 (Prime condition: attended-intact, unattended-intact, unprimed-intact) × 2 (Recording hemisphere: left, right) × 2 (Site: parietal P7/P8, occipital–temporal PO7/PO8) ANOVA which revealed a significant main effect of prime-condition [*F*(2,48) = 3.98; *p* = 0.025], that did not interact with hemisphere or site (*F*’s < 0.4; *p*’s > 0.4). Follow-up analyses revealed significantly enhanced N1 amplitudes (ERP priming) triggered to attended-intact probes in the contrast with unprimed-intact [*F*(1,24) = 7.97; *p* = 0.009], see **Figures 3** and **5A**, whilst ERPs to unattended-intact did not differ to unprimed (*F*’s < 2; *p*’s > 0.15). The second 3 × 2 × 2 ANOVA on N1 mean amplitudes triggered to probes preceded by split primes revealed a non-significant effect of prime-condition (attended-split, unattended-split, unprimed-split) [*F*(2,48) = 1.49; *p* > 0.2], but a significant interaction between prime-condition and electrode site [*F*(2,48) = 3.46; *p* = 0.041]. In neither the intact nor the split factorial ANOVA did prime-condition interact with recording hemisphere (all *p*’s > 0.88). Follow-up analyses confirmed a reliable ERP repetition effect, identified as an enhancement of N1 amplitudes triggered to attended-split trials in the contrast with unprimed-split trials, however, this priming effect was focal to left lateral electrode site P7 [*t*(24) = 2.383; *p* = 0.025]. N1 amplitudes at PO7, P8, and PO8 did not differ between attended split and unprimed split trials. Furthermore N1 amplitudes did not differ between unattended split and unprimed split^4^ at P7, P8, PO7, and PO8 (all *p*-values > 0.18)

Taken together the results suggest that attention is a prerequisite for posterior N1 (140–180 ms) ERP priming effects. Furthermore, priming for attended intact (same configuration) repeated probes was found at bilateral posterior electrode sites, whilst attended split (different configuration) trials produced ERP priming at left lateral posterior sites only. Interestingly, despite the lateralized pattern of N1 ERP priming effects for attended trials that depend on configuration of the preceding primes, follow-up contrasts showed N1 amplitudes did not differ between attended intact and attended split trials at either left [*t*(24) = 0.913; *p* = 0.37] or right lateral posterior electrodes [*t*(24) = 1.387; *p* = 0.18], see **Figure 5A**. Importantly, no posterior N1 ERP priming effects were found for unattended trials following either intact or split primes.

#### ERP Repetition Effects during P150 (140–180 ms)

Analyses of ERP repetition effects were focused on the P150 (140–180 ms) measured during the same time window as the posterior N1 but recorded at fronto-central sites (FCz and Cz). Similar to the previous analyses, two within-subjects ANOVAs were conducted on ERPs to probes preceded by either intact or split primes. The first 3 (Prime condition: attended-intact, unattended-intact, unprimed-intact) × 2 (Site: FCz, Cz) ANOVA revealed a significant effect of prime-condition [*F*(2,48) = 9.55; *p* < 0.001] that did not interact with electrode site [*F*(2,48) = 0.91; *p* > 0.40]. Follow-up analyses conducted separately on attended-intact and unattended-intact trials against unprimed-intact conditions revealed an enhanced P150 that reflected ERP effects of repetition for both attended-intact [*F*(1,24) = 14.65; *p* < 0.001] and unattended-intact trials [*F*(1,24) = 5.45; *p* < 0.03] (see **Figure 5B**); in neither contrast did prime-condition interact with electrode site (both *p*’s > 0.15).

A very different pattern of ERP repetition effects was found in the analyses for split trials at fronto-central electrodes during the P150. Results revealed no significant effect of prime-condition (attended-split, unattended-split, unprimed-split) [*F*(2,48) = 1.41; *p* > 0.25] or interaction between prime-condition and site [*F*(2,48) = 0.10; *p* > 0.90]. Follow up contrasts confirmed this result with the finding that P150 amplitudes did not discriminate significantly between attended-split and unprimed trials [*F*(1,24) = 3.87; *p* = 0.063] nor unattended split and unprimed [*F*(1,24) = 0.88; *p* = 0.35] furthermore, prime-condition did not interact with electrode site in either contrast (both *p*-values > 0.7). Taken together the results demonstrate that P150 amplitudes index ERP repetition effects for intact objects regardless of whether they had been at the focus of attention during the preceding prime trial (**Figure 5B**). Topographic scalp maps of ERP differences between significantly primed and unprimed trials in the 140–180 ms time interval are shown in **Figure 5C**.

#### ERP Effects of Attention and Part-Whole Configuration during N1/P150 (140–180 ms)

To assess the combined impact of attention and configuration on mean amplitudes triggered to probe objects, difference ERP waveforms were computed for each of the priming conditions during the N1/P150 (140–180) time interval. For example, ERPs on unprimed trials were subtracted from ERPs to each of the four repeated conditions (attended/intact; attended/split; unattended/intact; unattended/split). The four resulting difference ERPs (priming conditions) were subjected to factorial within-subjects ANOVA. First, a 2 (Attention: attended, unattended) × 2 (Configuration: intact, split) × 2 (Recording hemisphere: left, right) × 2 (Site: P7/P8, PO7/PO8) ANOVA on N1 amplitudes revealed no significant main effects of attention [*F*(1,24) = 1.91; *p* = 0.17] or configuration [*F*(1,24) = 0.39; *p* = 0.53], or interactions involving attention, configuration, hemisphere or site (*F*-values < 2; *p*’s > 0.3). Next, P150 (FCz, Cz) difference ERPs were computed for each of the four repeated conditions. Repeated measures ANOVA 2 (Attention: attended, unattended) × 2 (Configuration: intact, split) × 2 Site (FCz, Cz) showed no main effect of attention [*F*(1,24) = 2.96; *p* = 0.98] or configuration [*F*(1,24) = 0.96; *p* = 0.33]. There was no two-way interaction between attention and configuration and no three-way interaction with site (*F*-values < 8; *p*’s > 0.4). (See **Table 2**.)

**Table 2:** Summary of repetition effects according to time window and electrode location.

	N1/P150			N250r	
	Posterior Left	Posterior Right	Fronto-Central	Posterior Left	Posterior Right
Attended Intact	^∗^	^∗^	^∗^	^∗^	^∗^
Attended Split	^∗^	ns	ns	^∗^	^∗^
Unattended Intact	ns	ns	^∗^	ns	^∗^
Unattended Split	ns	ns	ns	ns	ns

*^∗^denotes significant (*p* < 0.05, Bonferroni corrected) ERP repetition effects (primed vs. unprimed)*.

#### ERP Repetition Effects during N250r (220–300 ms)

**FIGURE 6:**
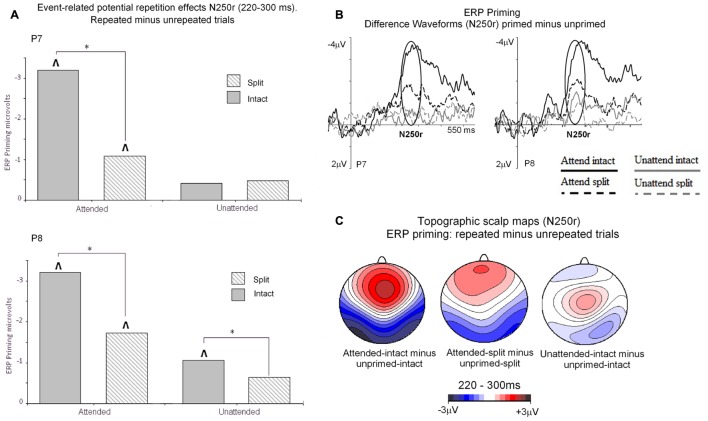
**(A)** N250r (220–180 ms) mean amplitudes obtained at posterior electrodes (P7/P8). Plotted separately and obtained by subtracting ERPs to unprimed (S2) trials from ERPs to repeated probes on attended or unattended trials for intact and split configuration objects. The ^∗^ denotes a significant difference between primed conditions, the upward arrow ˆ denotes significant N250r repetition effects (ˆ*p* < 0.05). (B) ERP difference waveforms recorded at lateral posterior electrodes P7 and P8 and obtained by subtracting ERPs to unprimed-intact objects from attended and unattended intact and ERPs to unprimed-split from ERPs to attended-split and unattended-split. The time-window of the N250r (220–300 ms) is highlighted. (C) Topographic scalp maps showing difference potentials obtained during the latency of the N250r (220–300 ms) after (S2) probe onset. Maps were computed by subtracting ERPs on unprimed-intact trials from ERPs to attended-intact, ERPs from unprimed-intact minus unattended-intact and ERPs to unprimed-split from attended-split probes. ERPs to unattended-split did not reliably differ to unprimed-split.

Event related potential repetition effects following latencies greater than 200 ms after probe onset (**Figures 3** and **4**) were found as enhanced negative amplitudes for repeated/primed as compared with unrepeated/unprimed trials (N250r). To fully assess the statistical significance of ERP repetition effects during the latency of the N250r (220–300 ms) two separate 3 (prime-condition) × 2 (hemisphere) × 2 (site) factorial ANOVAS were conducted for intact and split conditions separately. For trials with intact primes (attended-intact, unattended-intact and unprimed-intact), results revealed a significant main effect of prime-condition [*F*(2,48) = 43.85; *p* < 0.001] that marginally interacted with hemisphere [*F*(2,48) = 2.72; *p* = 0.07] but not with electrode site [*F*(2,48) = 0.52; *p* = 0.59]. Follow-up analyses confirmed the presence of reliable N250r (ERP priming) effects for attended-intact compared with unprimed at both right [*F*(1,24) = 70.36; *p* < 0.001] and left [*F*(1,24) = 56.61; *p* < 0.001] posterior sites. For unattended-intact trials the N250r differed to unprimed at right lateral posterior electrodes [*F*(1,24) = 8.37; *p* = 0.008] but not in the left sites (*F* < 1.38; *p* > 0.25). In neither analysis did prime-condition interact with electrode site (*p*’s > 0.1), see **Figure 6A**. The same analyses for split trials revealed a main effect of prime-condition [*F*(2,48) = 13.87; *p* < 0.001] that did not interact with hemisphere [*F*(2,48) = 0.21; *p* = 0.80] or electrode site [*F*(2,48) = 0.03; *p* = 0.96]. Follow-up analyses confirmed ERP priming on attended-split trials in the contrast with unprimed-split [*F*(1,24) = 37.96; *p* < 0.001] but demonstrated an absence of ERP priming during the N250r for unattended-split [*F*(1,24) = 2.39; *p* = 0.13]. To summarize, ERP repetition effects during the N250r were present at bilateral posterior electrode sites for attended-intact and attended-split probes, and at right lateral posterior sites for unattended-intact probes (**Figures 6A–C**).

#### ERP Effects of Attention and Part-Whole Configuration during N250r (220–300 ms)

Event related potential effects of attention and configuration were assessed by computing difference waveforms for repeated conditions (ERPs on repeated minus unrepeated trials). The resulting N250r difference ERPs were then subjected to a 2 (attention: attended, unattended) × 2 (configuration: intact, split) × 2 (recording hemisphere: left, right), × 2 (electrode: PO7/P7 vs. PO8/P8) repeated measures ANOVA. Results revealed significant main effects of attention [*F*(1,24) = 43.27; *p* < 0.001] and configuration [*F*(1,24) = 4.96; *p* < 0.036] moderated by a significant interaction between attention and configuration [*F*(1,24) = 7.41, *p* < 0.013]. There were no interactions involving attention or configuration with the factors recording hemisphere or site (*F*’s < 2.2; *p*’s > 0.15). Follow-up analyses for attended trials revealed significantly enhanced N250r amplitudes for attended-intact compared with attended-split at both left and right posterior sites (*t*’s > 3; *p*’s < 0.003). Whereas N250r amplitudes to unattended-intact trials did not differ from unattended-split at either left or right posterior sites (all *p* > 0.7) In summary, N250r amplitudes at lateral posterior electrodes were significantly different on attended-intact compared with attended-split trials, and unattended-intact did not differ to unattended-split trials. (See **Table 2**.)

## Discussion

The present study revealed behavioral short-term repetition priming of the naming of intact objects from previously attended objects in both intact and split views. By contrast, repetition priming from unattended objects was strictly view-dependent. Similar repetition effects were found using ERP measures, during time windows 140–180 ms (associated with the N1/P150) and in particular in the time window 220–300 ms (N250r) (see **Table 2**). For occipital–temporal sites enhanced amplitudes during the N1 time-window revealed very early (less than 200 ms after onset of the probe object) repetition effects. The magnitude of N1 ERP repetition effects for attended trials found at left posterior sites did not differ for attended-intact and attended-split objects, a result that likely reflects the activation of attention sensitive neurons that code for view-independent features of objects. However, ERPs in this early time window at right posterior sites revealed priming for attended-intact probes only. Importantly, during the same time window (140–180 ms) an enhanced P150 recorded over central and fronto-central scalp sites demonstrated reliable ERP repetition effects for attended and unattended objects repeated in the same intact configuration. Crucially, P150 ERP priming effects were not revealed for split conditions, which suggest that neural generators that contribute to the P150 reflect the activation of ‘view sensitive’ cells that operate independent of attention. The functional pattern of early repetition-sensitive ERPs provides evidence for temporally overlapping neural mechanisms differentially sensitive to the allocation of spatial attention and the constancy of image features across initial and repeated presentations. Important to note is that early ERP priming effects for repeated same-image objects do not reflect image priming afforded by simple pixel-by-pixel overlap, as objects were repeated at different locations in the visual field between first and second presentation on all trials.

During the time window between 220 and 300 ms (N250r) following the onset of probe objects pronounced ERP object repetition effects were observed as more negative-going amplitudes at occipital–temporal scalp sites in all but the unattended-split condition, with attended-intact images showing more enhanced repetition effects than split ones, although for unattended-intact conditions this ERP priming was only observed in the right hemisphere.

The behavioral priming effects (in the form of faster covert naming) and ERP repetition effects (during the N1/P150 and N250r time windows) mirror previous findings in fMRI studies (Thoma and Henson, 2011), and also mirror behavioral results found with overt naming (e.g., Stankiewicz et al., 1998; Thoma et al., 2004). Our main hypotheses for ERP effects derived from the Hummel (2001) model and were primarily focused on the N250r time window, because previous reports of ERP repetition effects during a time window between 200 and 300 ms at posterior scalp sites are thought to reflect the comparison between structural representations [within a modality-specific, pre-semantic, perceptual representation system (PRS)] and stored representations (Zhang et al., 1997; Martin-Loeches et al., 2005). ERP repetition effects were larger for intact than split conditions in attended conditions, similar to behavioral priming^5^. Importantly, as predicted, repetition effects were obtained even for probes that had been unattended in the preceding prime trial, but only when the related prime objects were shown in an intact configuration (although for N250r the differences between unattended intact and split images did not reach significance). Across the two time-windows this pattern of priming and repetition effects is generally consistent with the concept of two qualitatively different parallel processing routes mediating object recognition: an analytic route, part-based and dependent on attentional resources, involving representations for the recognition of unfamiliar views of familiar objects (e.g., split conditions), and a holistic route, view-dependent and automatic, in which view-based priming works without visual attention (Hummel, 2001).

Is it possible that the repetition effects for unattended objects may have resulted from residual attentional processing? Arguably, the manipulation of spatial cueing may have not completely prevented a spill-over (Lavie et al., 2004) or leakage (Lachter et al., 2004) of attention to the uncued object in the prime display. However, there is little evidence to suspect leakage (or more systematic spill-over) of attention when using the spatial cueing paradigm: Thoma and Davidoff (2006) used the last trial in one of their experiments as a catch-trial and asked participants whether they could name the unattended object. None of the 28 participants could do this correctly, indicating that there was no evidence for attentional processing of uncued prime objects.

Importantly, the repetition effects reported here—both behavioral as well as their ERP correlates—indicate a pattern of priming that cannot be predicted by either strictly part-based (structural) accounts of object recognition nor strictly view-based models alone (see the predictions in **Figure 1**). We will discuss the implications of this outcome after reviewing how our data compare to previous EEG studies on object recognition.

### ERP Effects for Object Repetition Prior to 200 ms after Probe Onset

The current results revealed early repetition effects as an enhanced occipital–temporal N1 for attended objects in intact and split views at left posterior sites and at right electrodes only for attended-intact conditions. An accompanying P150 during the same time window at central and fronto-central scalp sites revealed ERP priming for intact primes, both attended and unattended, but not probe trials preceded by split primes split primes. These results are in line with reports of early object-sensitive activation before 200 ms (see Schendan and Lucia, 2009, for a brief overview). The posterior N1 component has been implicated in the structural encoding of global configurations of objects (Doniger et al., 2001; Soldan et al., 2006), early feature processing in visual cortex (Eddy et al., 2006), implicit visual categorization of objects (Vogel and Luck, 2000), perceptual representation of object shape (Lloyd-Jones et al., 2012), and the implicit retrieval of perceptual features (Guillaume et al., 2009). Studies that have previously reported early ERP components (∼50–200 ms post stimulus onset) have considered these to reflect early categorization processes that route information to neural regions specialized for the processing of specific information such as faces or objects (Clark et al., 1995). Furthermore, early ERP components prior to 200 ms following the onset of a stimulus have been proposed to reflect activation corresponding to low-level stages of visual analysis (Schendan and Kutas, 2003; Henson et al., 2004; Itier and Taylor, 2004; Trenner et al., 2004) and perceptual processing (Schendan et al., 1998). Henson et al. (2004) noted that posterior ERP priming effects during the latency of the N1 were observed with a similar topography to later N250r repetition sensitive ERPs and likely reflect the earlier onset of repetition sensitive neural generators involved in later ERP repetition effects during latencies associated with an N250r.

There is evidence that the N1 component is involved in the structural analysis of fragmented images (Doniger et al., 2001) and encoding of global configurations (Eimer, 2000; Itier and Taylor, 2002, 2004). Soldan et al. (2006) reported ERP repetition effects during the latency of the N1 (see also Eddy et al., 2006) for objects with a coherent global structure, but not for impossible objects that comprise of parts. Schendan and Kutas (2003) reported a P150 largest over the vertex using an implicit long term object repetition paradigm. The authors noted that this early effect was ‘form specific’ in that ERP effects of repetition were largest for objects repeated in the same as compared with different views. Moreover, Harris et al. (2009) found implicit object repetition effects as a vertex P150 insensitive to the level of processing during encoding, which the authors suggested reflects an early stage of visuo-perceptual processing.

Thus, the results of the current study clarify the time course and functional characteristics of ERP repetition priming effects as they emerge at central and posterior scalp sites, revealing distinct patterns of viewpoint-invariance at left posterior scalp sites for attended objects, and viewpoint-dependence at central scalp sites. These results lend some support to the notion of a fast, view-dependent representation of object shape, with the functional properties of ERP priming at central scalp sites (P150) being in line with this model (Hummel, 2001). However, our results also showed that, during the same time window as the P150, temporally overlapping ERP priming effects for previously attended probes at left posterior scalp sites (N1) were present, with properties of invariance that revealed ERP priming insensitive to changes in image features between initial (prime) and repeated (probe) trials. The results of the current study provide support for a neural model built on rapid and temporally overlapping representations with functional properties (holistic and analytic) that can accommodate hybrid models of object recognition as that proposed by Hummel (2001).

### Comparison with Previous ERP Studies of Object Repetition Effects – N250r

Similar to our results previous research has found that the most pronounced ERP repetition effects are observed during latencies after 200 ms post stimulus onset (Rugg and Coles, 1995; Henson et al., 2004; Martin-Loeches et al., 2005) and can last up to 600 ms (Zhang et al., 1997; Eddy et al., 2006). The N250r has been mainly reported in studies investigating the repetition of faces and has been referred to as an early repetition effect (ERE; Schweinberger et al., 1995) as distinguished from the later repetition effect (LRE) or N400 (Martin-Loeches et al., 2005). However, ERP effects of object repetition have also been reported at posterior scalp sites during the latency 200–300 ms with similar temporal and spatial characteristics to that of the N250r (Zhang et al., 1997; Doniger et al., 2001; Henson et al., 2004; Martin-Loeches et al., 2005).

Schendan and Kutas (2007) have investigated long-term priming (tapping implicit memory) for objects that were intact or fragmented (that is, small line segments were removed from intact line drawings of objects in familiar views) in the study phase, and then presented in the same fragmented or differently fragmented (same view but non-overlapping line segments) version. Using an object categorization task they found (P200) repetition effects in occipital–temporal areas for fragmented study-test pairs (even when fragments did not overlap), but not for intact-fragmented pairs. Martin-Loeches et al. (2005) suggest that the N250r reflects a structural stage of processing, which may be modality-specific because no overlap in the spatial distribution of ERP priming effects was found when an object picture was repeated (object–object trials) compared to when an object name was repeated (object–name trials). The authors suggested that the N250r is an index of a SDS related to the concept of a sub-system of the PRS as proposed by Zhang et al. (1997). Our study clearly adds to this literature in showing that at least one neural component of object recognition is not view-specific, but depends on visual attention.

Anterior positive-going deflections after repetitions have been attributed to semantic or conceptual priming of objects (McPherson and Holcomb, 1999; Henson et al., 2004). Schendan and Kutas (2007; see also Schendan and Lucia, 2009) associate the fronto-central N300 with the process of object selection, meaning the matching of incoming percept and stored object model, and attribute its source to occipito-temporal generators. However, our current results point to object model selection much earlier, namely in the N250r time window, because we find reliable ERP repetition effects after previous presentation of split objects at bilateral occipital–temporal electrodes, but also during the earlier time window of the N1 (although less sustained than the N250r and more focal, because limited to left lateral posterior electrodes).

In general, our results are broadly consistent with previous findings on object repetition for attended images. The N250r (reported with similar characteristics to Ncl, see Doniger et al., 2000, 2001) in particular seems to indicate largely perceptual processing of object shape. Importantly, the present results extend previous research in showing that split images elicit repetition effects, which cannot be attributed to activation of stored views. This study is also to our knowledge the first to report largely view-dependent short-term repetition effects for spatially unattended objects.

In particular, our results extend previous findings that found early occipital–temporal effects of object repetition, which have shown early repetition components that may not depend on exact image-overlap (Schendan and Kutas, 2007) but maybe nevertheless be overall largely view-dependent (Schendan and Kutas, 2003). As described in the Introduction, the view-manipulations used, for example, in Schendan and Kutas’ (2003) study, in which they found linear effects of view changes for repetition effects, are for practical and theoretical reasons not well placed to test between models of object recognition. Therefore, while our results are in principle in agreement with previous findings of early view-dependent repetition effects, our conclusions are different in regards to the mechanisms underlying object representations, in particular the notion that mental rotation or view interpolation may compensate between views (Schendan and Kutas, 2003, 2007). Instead, the present unique combination of the factors attention and view-change allows a more direct test of whether visual attention has a qualitative influence on the nature of object representation as expressed by different ERP repetition effects, which we discuss in the next section.

### The Role of Attention in ERP Object Repetition Effects

Modulations in brain activation reported during latencies after 90 ms post stimulus onset have been localized to extrastriate cortical regions and have demonstrated increased activation for attended inputs compared with unattended (see Di Russo et al., 2003, for a discussion). Eddy et al. (2006) used a masked object priming paradigm in which pictures of common objects were presented (centrally) for short durations (50 ms) and were immediately followed by a mask. Subsequent probes were either the same or a different object. The findings of this study suggested that, even though prime objects were presented subliminally and therefore processed with no or little attention, repetition effects indicated a high level of visual processing, with an early effect of repetition evident as enhanced positive amplitudes at anterior and central sites being polarity-reversed posteriorly for the same objects from 100 to 190 ms. In particular during early components, ERPs are similar for both attended and unattended stimuli (Martínez et al., 2001) and during the early C1/P1 (50–90 ms), waveforms are unaffected by attention whereas longer latency deflections (150–225 ms) are seen to diverge for attended and unattended inputs (Eddy et al., 2006). Other studies—such as in dual task paradigms when attention is deployed elsewhere—also suggested that a minimum of processing resources is sufficient for a level of coarse categorization to take place. Thus, the current results confirm and extend the previous findings with ERP components to unattended stimuli, by showing that, even in the case when objects are spatially uncued, there are occipital–temporal (and fronto-central) repetition effects indicating object-form specific processing. However, unlike for attended objects, we observe that repetition effects for unattended objects are view-dependent.

### Implications for Theories of Object Recognition

The current data indicate that object repetition effects are highly sensitive to the manipulation of view (here intact versus split configurations), an observation that is generally consistent with theories that explain object constancy via a mechanism of view transformations or interpolations across 2D views (Ullman, 1989, 1998; Poggio and Edelman, 1990; Bülthoff and Edelman, 1992; Tarr, 1995; Tarr and Gauthier, 1998) or by assuming distributed neural representations across view-tuned neuronal groups (Perrett et al., 1998; see Peissig and Tarr, 2007, for a review). However, the prediction of view-dependent recognition performance is not unique to these models, and structural description theories are also able to account for certain view-specific effects (Hummel and Biederman, 1992; Biederman and Gerhardstein, 1993; Biederman, 2000). In fact, repetition effects for split images cannot be readily explained by strictly view-based accounts, as a split image of a horse is by definition not a view (in the sense of a holistic representation; see Hummel, 2001) if it has never been seen (and therefore been encoded) in such a configuration before (Thoma et al., 2004).

To account for structural properties of object recognition some view-based models have proposed templates for object “fragments” rather than view-based templates for whole objects (e.g., Edelman and Intrator, 2000, 2003; Ullman, 2007). These fragments are pictorial 2D features that underlie object recognition, but unlike generic parts in structural descriptions they cannot be freely combined with each other. However, the establishment of fragments requires multiple saccades from observers (Edelman and Intrator, 2000, 2003) to allow their binding to specific locations in the visual field (termed ‘what+where’ coding; see Edelman and Intrator, 2000, 2003). Because of these properties, fragment accounts cannot predict the observed visual priming from split images (here viewed during a single saccade) to their intact counterparts (shown at a different location between prime and probe display) as found in the current and similar studies (see Hummel, 2001, and Thoma et al., 2004, for more in-depth discussions of fragment and feature accounts).

The present evidence for repetition effects from split objects also provides only limited support for pure part-based accounts of object representations (e.g., Hummel and Biederman, 1992) because the current ERP results also indicate a substantial view-specific representational component. This notion could be challenged by the conjecture that splitting an object image may disrupt some spatial relations within the structural description, and that processing the two halves may require additional processing capacity, which in turn diminishes subsequent priming. However, it has been previously found that splitting object images overall seems to have no or minimal effects on structural descriptions and that a split (attended) object primes its identical split self just as much as intact images prime each other (Thoma et al., 2004, Experiment 3). Therefore, our conclusion is similar to those of Stankiewicz et al. (1998) and Thoma et al. (2004), which is that neither part-based (structural) theories of object recognition nor view-based models alone are able to account for the obtained pattern of behavioral and ERP repetition effects. Instead, the current ERP data are the first supporting an account of object recognition that postulates the involvement of both holistic (automatic and view-specific) and analytic (part-based) representations in mediating object recognition (e.g., Hummel, 2001).

The hybrid model (**Figure 1**) predicted repetition effects indicating shape processing that can compensate for configurational changes, while, at the same time, repetition effects indicating strong automatic view-dependent processing in both attended and unattended conditions. The current behavioral and ERP priming effects are broadly consistent with the hybrid model’s assumption that a previously attended probe object in an intact view contributes input from both analytic as well as holistic (view-dependent) processing to priming, whereas a previously attended probe object in a split view only contributes the input of the analytic shape processing route to priming. The behavioral priming results and the repetition effects observed in the right occipital–temporal electrodes in the N250r not only mirror closely previous behavioral and imaging data (Thoma et al., 2004; Thoma and Henson, 2011), but also reflect the simulations of the hybrid model’s computational instantiation (JIM.3; Hummel, 2001).

However, at least one aspect of the results does not fully fit with the current instantiation of the hybrid model. View-specific repetition effects were overall greater from intact than from split primes in the attended conditions than in the unattended conditions, as indicated by the significant interaction in the N250r window. Future investigations may clarify a further observation that generally fits with the hybrid model: the holistic route is supposed to build rapid representations relying on familiar patterns of 2D surfaces (constituting views). Indeed we found early repetition effects for probe objects around the N1/P150, which seem to fit with the hybrid models’ notion of a fast, holistic route of object recognition, because ERP repetition effects were not only found for attended-intact objects, but also for unattended objects in intact views (at central electrodes). Thus, overall we found strong neural evidence for a view-based component that is established rapidly, as proposed by the hybrid model.

## Conclusion

The current findings support and extend previous observations of ERP studies showing (N250r) occipital–temporal repetition effects for objects in identical views even when they had been previously spatially unattended in a prime display. At the same time we observed repetition effects for novel views of familiar objects in early time windows (<200 ms post stimulus onset) indicating that view-independent object model selection begins earlier than had been previously suggested by ERPs (e.g., Schendan and Kutas, 2007). These repetition effects were largely consistent with behavioral priming effects and, taken together with previous behavioral and imaging work, broadly support the notion that object recognition includes both analytic and holistic shape processing, with the analytic route more dependent on visual attention. Future research should establish whether the holistic components rely solely on automatic processing, for example, does the representation of holistic information reflect the activation of different neural generators to those involved in analytic processing, and whether our results hold up for other view-transformations.

## Author Contributions

All authors listed, have made substantial, direct and intellectual contribution to the work, and approved it for publication.

## Conflict of Interest Statement

The authors declare that the research was conducted in the absence of any commercial or financial relationships that could be construed as a potential conflict of interest. The reviewer JH declared a shared affiliation, though no other collaboration, with one of the authors AR-K to the handling Editor, who ensured that the process nevertheless met the standards of a fair and objective review.

## Footnotes

1 The polarity and whether repetition sensitive ERPs are found as an enhancement or reduction in amplitude often depend on factors such as task complexity, whether short or long term repetition processes are recruited and importantly whether explicit recollection or recognition of initial learning phase is a task requirement during encoding (Bentin and McCarthy, 1994; Penney et al., 2000).

2 One problem with traditional manipulations of object viewpoint is that these view-changes often confound changes in the structural description: picture-plane rotations usually perturb the spatial relations between parts, whereas depth-rotations may cause parts to be hidden or novel parts to be revealed (Thoma and Davidoff, 2006). An intact and split image of an object (e.g., a horse) are completely different holistic representations (features/parts are linked to different locations, Hummel, 2001), yet they constitute a very similar structural representation (note that spatial relations “left-of” and “right-of” are not coded differently in structural description models, see Hummel and Biederman, 1992).

3 http://www.biosemi.com/faq/cms&drl.htm

4 Although there was no main effect or interaction involving the factor hemisphere, we analyzed the electrodes for each side because Thoma and Henson (2011) observed strong lateralization for repetition effects in left temporal (attended) and right parietal (unattended) cortex. Further, the equivalent ERP analysis of the N250 time window —reported below—do show an interaction with hemisphere.

5 One possible criticism of this conclusion is that, because intact and split prime images were always followed by intact probes, it is unremarkable that behavioral priming effects are greater for intact conditions. However, previous work has shown that while an attended split object primed its identical split self just as much as an intact image primes its identical self, this effect is not found for unattended images (Thoma et al., 2004, Experiment 3). In other words, there is not even picture-to-picture priming for split probe images when they have been uncued (as splits) in the prime display (see also similar results for upside-down objects, see Thoma and Davidoff, 2007).
